# Increased Levels of C-C Chemokine RANTES in Asbestos Exposed Workers and in Malignant Mesothelioma Patients from an Hyperendemic Area

**DOI:** 10.1371/journal.pone.0104848

**Published:** 2014-08-27

**Authors:** Manola Comar, Nunzia Zanotta, Alessandra Bonotti, Mauro Tognon, Corrado Negro, Alfonso Cristaudo, Massimo Bovenzi

**Affiliations:** 1 Institute for Maternal and Child Health-IRCCS “Burlo Garofolo”–Trieste, Trieste, Italy; 2 Clinical Unit of Hygiene, Department of Medical Sciences, University of Trieste, Trieste, Italy; 3 Division of Occupational & Preventive Medicine, University Hospital of Pisa, Pisa, Italy; 4 Department of Morphology, Surgery and Experimental Medicine, Section of Pathology, Oncology and Experimental Biology, School of Medicine, University of Ferrara, Ferrara, Italy; 5 Clinical Unit of Occupational Medicine, Department of Medical Sciences, University of Trieste, Trieste, Italy; University of Leuven, Rega Institute, Belgium

## Abstract

**Background:**

Asbestos-induced mesothelial inflammatory processes are thought to be the basic mechanisms underlying Malignant Mesothelioma (MM) development. Detection of MM often occurs at late stage due to the long and unpredictable latent period and the low incidence in asbestos exposed individuals. The aim of this study was to investigate early immunological biomarkers to characterize the prognostic profile of a possible asbestos-induced disease, in subjects from a MM hyperendemic area.

**Methods:**

The Luminex Multiplex Panel Technology was used for the simultaneous measurement of serum levels of a large panel of 47 analytes, including cytokines and growth factors, from workers previously exposed to asbestos (Asb-workers), asbestos-induced MM patients and healthy subjects. In addition, to explore the influence on serum cytokines profile exerted by SV40 infection, a cofactor in MM development, a quantitative real time PCR was performed for sequences detection in the N-terminal and intronic regions of the SV40 Tag gene. Statistical analysis was done by means of the Mann-Whitney test and the Kruskall-Wallis test for variance analysis.

**Results:**

A variety of 25 cytokines linked to pulmonary inflammation and tumor development were found significantly associated with Asb-workers and MM patients compared with healthy controls. A specific pattern of cytokines were found highly expressed in Asb-workers: IFN-*alpha* (p<0.05), EOTAXIN (p<0.01), RANTES (p<0.001), and in MM patients: IL-12(p40), IL-3, IL-1 *alpha*, MCP-3, *beta*-NGF, TNF-*beta*, RANTES (p<0.001). Notably, the chemokine RANTES measured the highest serum level showing an increased gradient of concentration from healthy subjects to Asb-workers and MM patients (p<0.001), independently of SV40 infection.

**Conclusion:**

This study shows that, in subjects from an hyperendemic area for MM, the C-C chemokine RANTES is associated with the exposure to asbestos fibres. If validated in larger samples, this factor could have the potential to be a critical biomarker for MM prognosis as recently reported for breast tumor.

## Background

Human Malignant Mesothelioma (MM) is an aggressive tumor with an increased mortality rate that arises most often from the mesothelial cells of the pleura (90%), peritoneum and, occasionally, the pericardium [1]–[5].

The association of mesothelioma with asbestos exposure is well established, with an aetiological fraction above 80% [6], [7]. Crocidolite asbestos is considered to be most pathogenic of the several asbestos types in the induction of MM, although the World Health Organisation (WHO) has established that all types of asbestos fibers can cause cancer in humans [6]–[9].

Cooperation of epigenetic events including host genetic factors and carcinogens including virus infection, mainly SV40, has been also suggested for the onset of asbestos related malignancy [10]–[13]. Particularly, the direct involvement of the oncogenic virus SV40 in the growth and tumorigenicity of mesothelial cells, subordinate to insulin-like growth factor-1 release, has been documented and associated with a shorter survival of MM patients [14], [15].

Mesothelial inflammatory process is thought to be the basic mechanism underlying the pathobiology of MM. It has been described that a long inflammatory response regulated by mesothelial cells contributes to initiation, promotion and progression into MM [6], [16]–[19]. Most research regarding the role of inflammation in asbestos-associated diseases has focused on immune cellular response including the first cell type accumulating at sites of initial deposition of inhaled asbestos fibres. After injury, mesothelial cells can recruit neutrophils, monocytes and lymphocytes by producing chemokines and cytokines which induce mesothelial cells to release growth factors with paracrine functions [20]–[25]. Recent studies, on human mesothelial cells, support a model where an autocrine loop is perpetrated by fibres–induced inflammasone NLRP3 priming and activation, with the subsequent increased transcription activity of proinflammatory growth factors [26]–[29].

Several interesting candidates have been disclosed by a wide range of investigations carried out to identify soluble markers characterizing the pathobiology of MM. Among these molecules, the pro-inflammatory cytokines IL1-*beta*, IL-6, IL-8, the vascular endothelial growth factor (VEGF) promoting tumor angiogenesis, the hepatocyte growth factor (HGF) which stimulates cell migration and tumor invasiveness, the transforming growth factor beta (TGF-β) implicated in tumor growth, the platelet-derived growth factor (PDGF) which regulates MM cells proliferation and the tumor necrosis factor alpha (TNF-α), have been found more frequently over-expressed in the microenvironment of mesothelial cells during neoplastic transformation [21], [23], [24].

MM represents a highly aggressive tumor with poor prognosis due to the long and unpredictable latent period and the low incidence in chronically asbestos exposed individuals who develop the disease. The identification of a reliable sentinel biomarkers able to select long-term exposed subjects at high risk for MM, could represent a key point for the clinical management of these patients and for further studies on the immunological pathways influencing MM pathogenesis. To date, there is no evidence of effective prognostic biomarker associated to asbestos exposure.

On this basis, in order to improve our knowledge about the inflammatory process involved in the response to asbestos fibers, we were interested in exploring the immunological profile, including a large panel of cytokines (n = 47), in workers previously exposed to asbestos but free from MM disease (Asb-workers) and in asbestos exposed patients with MM compared with healthy individuals (control group) from an Italian MM hyperendemic area, identified as a cluster of the disease because of the massive use of asbestos in dockyards and shipyards in the past.

Moreover, in this study, SV40 infection, considered a cofactor in MM development, was additionally taken into consideration in the analysis of the expressed cytokines profile.

## Materials and Methods

### Ethical statement

The study was approved by the local Ethic Committee of the University Hospital “Ospedali Riuniti di Trieste” and informed consent was obtained from each participant in accordance with the principles outlined in the Declaration of Helsinki.

### Subjects

The subjects enrolled in this research were recruited from the Clinical Unit of Occupational Medicine of the University of Trieste, Italy, in the period 2010–2013. Information on asbestos exposure, based on standardized guidelines, was ascertained using the records of the local Mesothelioma Registry, affiliated to the National Mesothelioma Registry [30].

The enrolled Asb-workers, (n = 15; mean age of 50.6 years-range 48–55 years), were non-smokers and had an averaged occupational history of asbestos exposure of 25 years. At the radiological examinations, 3 subjects showed pleural plaques, 1 had pulmonary fibrosis, and 11 was free from lung or pleural alterations. Age-and height-adjusted spirometric data showed that pleural plaques were not associated with a loss of pulmonary function. The subjects suffered from MM (n = 15, mean age 69 years, range 55–85 years), were non-smokers, diagnosed with epithelioid malignant pleural mesothelioma, free from pre-operative chemotherapy and radiotherapy and no kind of diagnostic workup or treatment was provided to patients during the course of this study.

The diagnosis and stage of the MM was based on surgical pleural biopsy according to the World Health Organization criteria [30]. Clinical information including histologic diagnosis was obtained from pathology reports.

The healthy control group consisted of 13 healthy non-smokers volunteers, (mean age 63.3 years, range 46–75 years), with no hemathological evidence of autoimmune disorders (negative for Ab anti extracted nucleus and Ab anti DNA), and without findings of pulmonary dysfunction at the time of enrolment.

### Samples and DNA extraction

Following institutional approval and written informed consent, blood and serum samples were obtained from the 43 enrolled subjects. Whole blood of each subject was collected in a covered test tube without anticoagulants and allowed to clot by leaving it undisturbed at room temperature for 20 minutes. Clot was then removed by centrifuging at 1500×g for 10 minutes in a refrigerated centrifuge. Following centrifugation, the serum was immediately transferred into a clean polypropylene tube. All the serum were maintained at −80°C until cytokines analysis.

DNA was isolated from 500 µl of total blood using the automated extractor NucliSENS EasyMAG (BioMerieux, Durham, NC) following the generic protocol without modification, eluted in 25 µl and conserved at −20°C.

### Real time quantitative polymerase chain reaction for SV40

A multiple TaqMan real-time PCR assay (Q-PCR) was designed to simultaneously quantify approximately 100 bp in the conserved N-terminal region of the large T antigen (Tag) coding region of SV40 and 80 bp of the reference human β-globin gene, with a lowest limit of detection for both targets of 10 copies/reaction. In brief, for each reaction run, 10 µl of clinical DNA sample and 10 µl of the specific standard scale dilution (from 10^7^ to 10^0^ copies) detecting both SV40 and β-globin sequences were added to a final volume of 50 µl of reaction-mix and run in triplicate following the manufacture's instruction (RT Polyoma Panel kit, Eurospital Spa, Trieste, Italy).

An additional set of primers, SVINTfor 5-AAGTAAGGTTCCTTCACAAAG-3 and SVINTrev 5-AACTGAGGTATTTGCTTCTTC-3, amplifying a 235-bp intronic portion of the SV40 Tag gene, were additionally used as confirmatory test for SV40 detection. These primers are considered to be at low risk for false-positive results due to putative contamination by laboratory plasmids containing SV40. The PCR reaction was performed for 40 cycles with the profile: 15 min at 94°C, 45 s at 94°C, 45 s at 60°C, 1 min at 72°C for 40 cycles [31].The amplification products were then migrated in 0.8% acrylamide gel and visualized under UV after ethidium bromide staining.

### Chemokines and cytokines analysis

The main outcome measures were the quantification of cytokine and growth factors concentrations in biological samples based on magnetic bead multiplex immunoassays (Bio-Plex, BIO-RAD Laboratories, Milano, Italy). Luminex multiplex panel technology was used for simultaneous measurement of a panel of 47 analytes including cytokines and growth factors (IL-1*β*, IL-1ra, IL-2, IL-4, IL-5, IL-6, IL-7, IL-8, IL-9, IL-10, IL-12(p70), IL-13, IL-15, IL-17, Eotaxin, Basic FGF, G-CSF, GM-CSF, IFN-*γ*, IP-10, MCP-1, MIP-1*α*, PDGF-BB, MIP-1*β*, RANTES, TNF-*β*, VEGF, IL-1*α*, IL-2R*α*, IL-3, IL-12(p40), IL-16, IL-18, CTACK, GRO-*α*, HGF, IFN-*α*2, LIF, MCP-3, M-CSF, MIF, MIG, *β*-NGF, SCF, SCGF-*β*, SDF-1*α*, TNF-*α*) which standard values were showed in Table 1.

**Table 1:** Luminex multiplex panel technology for simultaneous measurement of 47 analytes including cytokines and growth factors.

Official Symbol	Other Designations	std value (max-min) (pg/ml)
**IL-1α**	Interleukin 1, beta	33.971-2.0
**IL-1ra**	Interleukin-1 receptor antagonist	30.173-1.39
**IL-2**	Interleukin 2, TCGF, lymphokine	15.605-0.97
**IL-4**	Interleukin 4, B cell growth factor 1	3.628-0.23
**IL-5**	Interleukin 5, B-cell differentiation factor I; T-cell replacing factor	32.404-1.97
**IL-6**	Interleukin 6, B-cell differentiation factor; B-cell stimulatory factor 2	19.412-1.18
**IL-7**	Interleukin-7	35.672-2.2
**IL-8**	Interleukin 8, CXCL8, alveolar macrophage chemotactic factor I	27.965-1.68
**IL-9**	Interleukin 9, T-cell growth factor p40; cytokine P40	10.705-0.8
**IL-10**	Interleukin 10, T-cell growth inhibitory factor; cytokine synthesis inhibitory factor	28.093-1.78
**IL-12(p70)**	Interleukin-12 subunit p70, CLMF, NKSF	36.141-2.17
**IL-13**	Interleukin 13, ALRH, BHR1	32.271-1.95
**IL-15**	Interleukin 15	24.906-1.49
**IL-17A**	Interleukin 17, CTLA-8; cytotoxic T-lymphocyte-associated antigen 8	21.505-1.49
**Eotaxin**	C-C motif chemokine 11; eosinophil chemotactic protein	23.066-1.5
**FGF basic**	Basic fibroblast growth factor; heparin-binding growth factor 2	18.366-1.36
**G-CSF**	Granulocyte colony-stimulating factor	29.458-1.69
**GM-CSF**	Granulocyte-Macrophage Colony Stimulating Factor	13.381-1.7
**IFN-γ**	Interferon gamma	30.646-1.68
**IP-10**	CXCL10, 10 kDa interferon gamma-induced protein	32.749-2.12
**MCP-1(MCAF)**	CCL2, C-C motif chemokine 2; monocyte chemoattractant protein 1	32.735-1.48
**MIP-1α**	CCL3, C-C motif chemokine 3; G0/G1 switch regulatory protein 19-1	21.878-1.34
**PDGF-bb**	Platelet derived growth factor, isoform b	25.637-1.65
**MIP-1β**	CCL4, C-C motif chemokine 4; CC chemokine ligand 4	20.198-1.3
**RANTES**	CCL5, C-C motif chemokine 5; SIS-delta	25.450-1.63
**TNF-α**	Tumor necrosis factor, alfa	75.618-471
**VEGF**	Vascular endothelial growth factor	29.354-1.87
**IL-1α**	Interleukin 1alpha	25.358-1.56
**IL-2Ra**	Interleukin receptor alpha	14.038-0.9
**IL-3**	Interleukin 3	33.650-2.06
**IL-12p40**	Interleukin 12p40	40.474-2.5
**IL-16**	Interleukin 16	17.134-1.06
**IL-18**	Interleukin 18	18.021-1
**CTACK**	Cutaneous T cell-attracting chemokine	29.629-1.63
**GROα**	Growth-related oncogene alpha	30.764-2.16
**HGF**	Human growth factor	37.918-2.02
**IFN-α2**	Interferon alpha 2	5159-0.3
**LIF**	Leukemia inhibitory factor	10.786-0.72
**MCP-3**	Monocyte chemotactic protein 3	16.308-1.01
**M-CSF**	Macrophage-colony-stimulating factor	31.887-1.99
**MIF**	Macrophage migration Inhibitory factor	25.549-1.67
**MIG**	Monokine induced by interferon	48.909-3.01
**β-NGF**	Nerve growth factor beta	18.102-1.1
**SCF**	Stem cell factor	37.457-2.25
**SCGF-β**	Stem cell factor growth factor -b	159.243-11.71
**SDF-1α**	Stromal-cell derived factor alpha 1	19.351-1
**TNF-β**	Tumor necrosis factor-b	36.892-2.3

Briefly, 50 µl of diluted (1∶4) serum samples and reaction standards were added, in duplicate, to a 96 multiwells plate containing analyte beads followed by incubation for 30 minutes at room temperature. After washing, the antibody-biotin reporter was added and incubated for 10 minutes with streptavidin-phycoerythrin. The levels of the cytokines were determined using the Bio-Plex array reader (Luminex, Austin, TX). The Bio-Plex Manager software optimized the standard curves automatically and returned the reading data as Median Fluorescence Intensity (MFI) and concentration (pg/mL). An ELISA set (Quantikine ELISA-Human CCL5/Rantes immunoassay, RnD system, Minneapolis, MN) with a mean minimum detectable dose of 2.0 pg/ml was used as confirmatory test according to manufacturer's instruction. Sera was diluted 1∶4 and the amount of RANTES was determined by absorbance of the samples by comparing the standards at 450 nm using the ELISA reader.

## Statistical Analysis

Data analysis was performed with the softwares Stata (v. 13.1) and GraphPad Prism (v. 5). Continuous data were summarised with the median as a measure of central tendency and the quartiles measures of dispersion. The comparison between two groups was made by means of the Mann-Whitney test. The Kruskall-Wallis one-way analysis of variance was used to compare more than two groups. When a significant p-value was found (p<0.05), a multiple comparison test was used to determine which groups were different.

## Results

### Cytokines analysis in Asb-workers and MM patients

A total of forty-three serum samples from 15 MM patients, 15 Asb-workers and 13 healthy controls were explored by investigating a large panel of cytokines (n°47), for the evaluation of their immunological profile. Moreover, the detection of SV40 infection was performed on matched blood samples and analyzed with respect to the expressed cytokines pattern.

The results from the analysis of the cytokines profiles showed that a set of 26 soluble analytes were differentially expressed among the three groups of subjects including in the study, while the remaining 21 did not differ significantly. The levels of sera concentration of these cytokines, measured as pg/ml, and the grade of the statistical significance were represented in Table 2.

**Table 2:** Serum concentration of significative cytokines in MM patients, Abs-workers and healthy subjects.

Cytokines	MM Patients (n°15)	Abs-workers (n°15)	Healthy subjects (n°13)
**IL-1α** ***	67.37 (0.35–75.03)^f^ **^,^** h	0.35 (0.35–0.35)	0.01 (0.01–0.01)
**IL-2Ra** **	51.62 (45.79–68.29)^e^	18.69 (0.13–33.83)^b^	152.30 (126.10–192.24)
**IL-3** **	63.68 (0.46–66.63)^f^ **^,^** h	0.46 (0.46–0.46)	0.46 (0.46–0.46)
**IL-12p40** **	216.53 (1.17–229.62)^f^ **^,^** h	1.17 (1.17–1.17)	1.17 (1.17–1.17)
**Ctack** *	829.24 (133.79–1028.74)^h^	108.30 (89.08–120.14)	250.39 (172.92–384.29)
**GROα** **	13.95 (0.47–19.68)	0.47 (0.47–0.47)^c^	24.17 (15.39–30.41)
**HGF** **	37.87 (17.05–59.59)^f^	30.54 (30.43–30.64)^b^	228.98 (164.78–494.60)
**IFN-α2** *	0.09 (0.09–14.93)	9.98 (7.85–17.07)^a^	0.09 (0.09–0.09)
**MCP-3** ***	13.91 (0.18–15.01)^f^ **^,^** h	0.18 (0.18–0.18)	0.18 (0.18–0.18)
**M-CSF** ***	1.04 (0.44–2.18)^f^	0.44 (0.44–0.44)^b^	10.39 (9.34–12.50)
**MIF** *	2.33 (1.06–28.25)^d^	1.06 (1.06–1.06)^a^	33.40 (19.13–41.67)
**MIG** **	70.87 (23.73–518.81)^d^	432.49 (396.93–466.37)	1032.23 (162.00–1876.80)
**β-NGF** ***	36.51 (0.26–36.56)^f^ **^,^** h	0.26 (0.26–0.26)	0.26 (0.26–0.26)
**SCGF-β** ***	1386.14 (3.16–1782.43)^e^	3.16 (3.16–3.16)^c^	6059.88(4970.20–50221.58)
**SDF-1α** ***	3.33 (0.57–7.18)^f^	0.57 (0.57–0.57)^c^	20.92 (19.90–22.40)
**TNF-β** ***	20.00 (0.36–20.29)^f^ **^,^** g	0.36 (0.36–0.36)	0.36 (0.36–0.36)
**IL-4** ***	3.40 (2.49–5.57)^f^	1.90 (1.44–2.39)^c^	7.09 (6.70–7.59)
**IL-6** *	16.91 (11.32–34.11)^g^	6.06 (0.38–11.63)	10.63 (7.94–12.21)
**IL-8** ***	17.13 (13.63–24.24)^e^ **^,^** g	10.77 (0.40–12.48)^c^	27.10 (22.79–30.51)
**IL-17A** *	59.01 (28.85–93.80)	28.99 (0.44–29.87)^a^	96.08 (88.64–115.72)
**EOTAXIN** *	0.48 (0.48–87.25)^g^	139.62 (95.31–223.62)^b^	24.30 (0.48–31.20)
**G-CSF** *	19.80 (13.51–42.33)	12.91 (10.25–13.36)	39.80 (28.70–54.57)
**IFN-γ** **	169.01 (54.64–230.36)^h^	38.71 (19.24–45.01)^a^	135.47 (127.89–149.47)
**IP-10**	1432.13 (522.52–1909.76)	851.81 (631.41–279.92)	484.73 (380.98–842.63)
**MIP-1α** ***	2.76 (0.30–3.44)^f^	0.30 (0.30–0.30)^a^	7.10 (6.12–7.46)
**RANTES** ***	34539.00 (20035.98–34539.00)^f^	23004.57(18507.41–34539.0)^c^	0.52 (0.52–052)

Data are given as medians (quartiles) (pg/ml);

*p<0.05;

**p<0.01;

***p<0.001.

Abs-workers vs Healthy subjects:

a p<0.05;

b p<0.01;

c p<0.001.

MM patients vs Healthy subjects:

d p<0.05;

e p<0.01;

f p<0.001.

MM patients vs Abs-workers:

g p<0.05;

h p<0.01;

i p<0.001.

**Figure 1:**
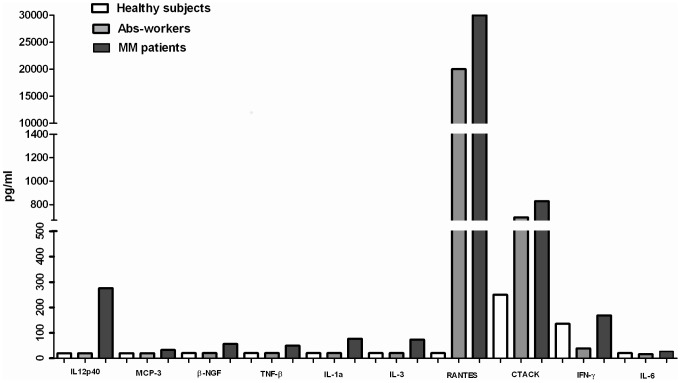
Immunological profile of significative up-regulated cytokines in sera of MM patients. A panel of 10 cytokines showed significantly higher levels of expression in serum of MM patients compared with Abs-workers and healthy subjects. IL-1*α*, IL-3, IL-12(p40), MCP-3, *β*-NGF, TNF-*β*, and RANTES (p<0.001) showed significantly higher levels in MM patients respect to controls. IL-6 (p<0.05) and CTACK, IFN-*γ* (p<0.01) were found over-expressed in MM patients when compared with the Asb-workers. The C-C chemokine RANTES showed the highest sera concentration documented by an increased gradient from healthy to Asb-workers and MM patients. The levels of each cytokines were expressed in pg/ml.

The cytokines profile was first analyzed by comparing the Asb-workers and the MM patients versus the healthy subjects (controls). Compared to the controls, sera from the Asb-workers contained significantly lower levels of a variety of cytokines, such as such as MIF, IFN-γ, MIP-1*α*, IL-17 (p<0.05) IL-2Ra, HGF, M-CSF, G-CSF (p<0.01); IL-4, IL-8, SDF-1*α*, GRO-*α*, SCGF-*β* (p<0.001); while 4 cytokines, including IFN-α (p<0.05), Eotaxin (p<0.01), IP10 (p<0.01) and RANTES (p<0.001) were significantly up-regulated. Regarding MM patients, a panel of 10 cytokines, MIF, MIG (p<0.05); IL-2Ra, SCGF-*β*, IL-8, (p<0.01); HGF, M-CSF, SDF-1*α*, IL-4, MIP-1*α* (p<0.001), was found down-regulated with respect to the control group. Conversely, 7 cytokines IL-1*α*, IL-3, IL-12(p40), MCP-3, *β*-NGF, TNF-*β*, and RANTES (p<0.001) showed significantly higher levels. In these patients an additional set of 3 pro-inflammatory factors, IL-6 (p<0.05) and CTACK, IFN-*γ* (p<0.01) were found over-expressed when compared with the Asb-workers (Figure 1) implying that a modulation of the inflammatory milieu was exerted during the pathologic process. Moreover, IP10, although statistically not significant (p = 0.09), was also found highly expressed in MM patients (Figure 1).

**Figure 2:**
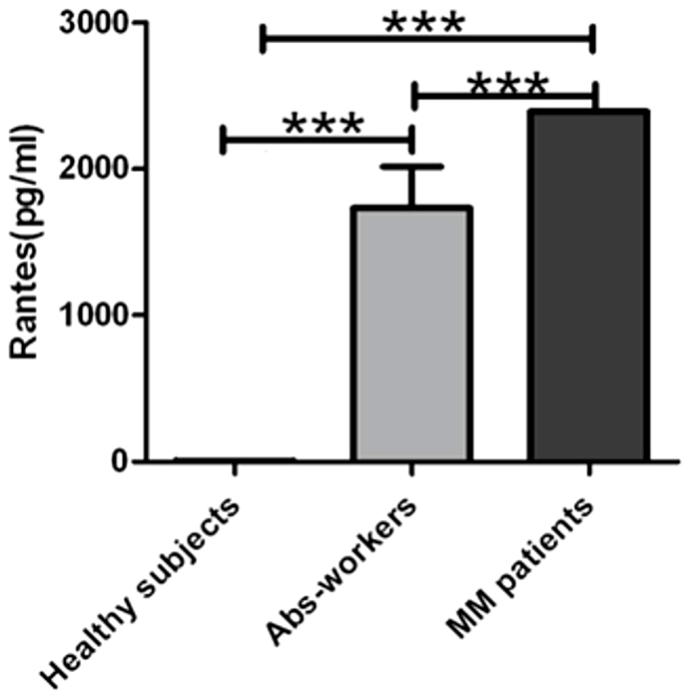
Rantes levels in sera of healthy subjects, Abs-workers and MM patients. Rantes levels in serum of MM patients compared with Abs-workers and healthy subjects using Elisa test. This chemokine showed significantly higher levels of expression in serum of MM patients (p<0,001), respect to other two group of subjects. The levels of chemokine were expressed in pg/ml. The comparison between the three groups was made by the Kruskall-Wallis one-way analysis of variance. The significant results are marked with asterisks: ***p<0.001.

Of note, among the above cited cytokines, the C-C chemokine RANTES showed the highest level of expression documented by an increased concentration gradient from the healthy subjects (0.52 pg/ml) to the Asb-workers (23004 pg/ml) and MM patients (34539 pg/ml) (p<0.001) suggesting a strong association of this growth factor with the exposure to asbestos fibres. This finding was additionally evaluated by a sandwich ELISA assay which results paralleled the trend of concentration previously detected. As showed in Figure 2, the RANTES levels were significally elevated in MM (2390.4 pg/ml) compared with Asb-workers (1590.44 pg/ml) and healthy subjects (3.55 pg/ml) (p<0.001).

**Figure 3:**
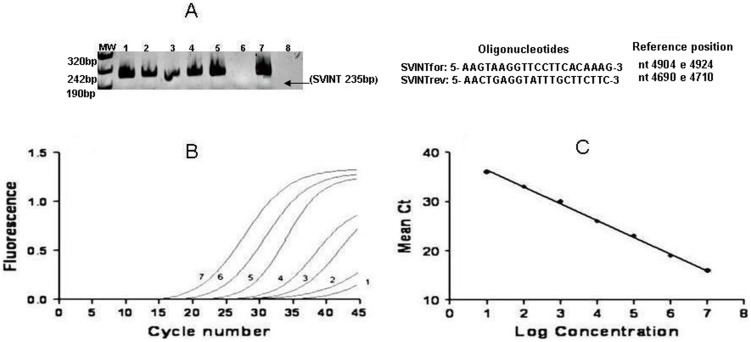
Real time PCR for SV40 detection in blood from Abs-workers, MM patients and Healthy subjects. (**A**) Acrylamide 0,8 gel electrophoresis of PCR-amplified sequence of the SV40 intronic regions of the Tag gene (set of primers SVINT, product size of 235 bp) stained by ethidium bromide. MW: Molecular weight markers. Lanes 1–5: DNA blood samples from MM patients found SV40 positive. Lane 6: DNA blood sample of a MM patient resulted SV40 negative. Lane 7: positive control represented by pBR322 plasmid containing the whole SV40 DNA wild-type strain. Lane 8: negative PCR reaction control. (**B**) SV40 amplification curves (from 10^0^ to 10^7^ copies/ul) of quantitative Real Time PCR. Fluorescence units are plotted against the cycle number for each standard dilution series while the log_10_ of the input copy number is indicated on the amplification plot. (**C**) Amplification standard curve for detection of unknown quantities of SV40 in blood samples. Standard curve was generated by plotting the observed threshold cycle, (CT) against the log_10_ of the input copy number of standard plasmid sequences in the conserved N-terminal region of the large Tag of SV40.

SV40 infection was ascertained only in 5 blood samples from MM patients, which tested positive for both intronic and N-terminal coding sequences of the SV40 Tag gene, with a viral DNA load from 2.4×10^2^ to 1.5×10^3^ copies/reaction (Figure 3).

**Figure 4:**
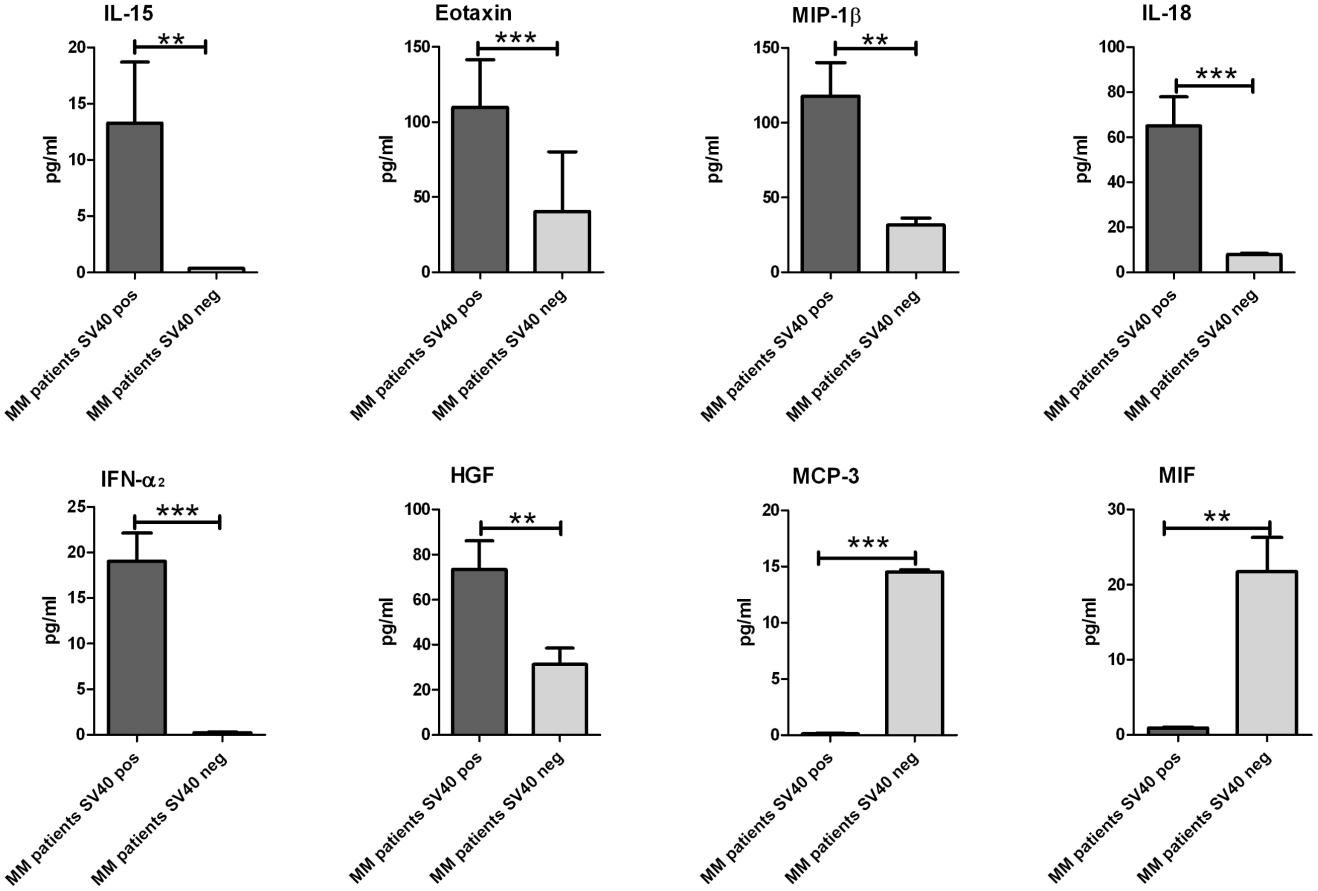
Expression of specific cytokines associated with SV40 infection in sera of MM patients. Median values of cytokines found down and up regulated in serum of SV40 positive MM patients with respect to SV40 negative MM patients. IL-15, EOTAXIN, MIP-1β, IL-18, IFN-α2, and HGF were up-regulated (p<0.01) whereas MCP-3 and MIF (p<0.01) were down regulated. The comparison between the two groups was made by means of the Mann-Whitney non-parametric Student's t-test. The significant results are marked with asterisks: *p<0.05, **p<0.01, ***p<0.001.

The cytokines profile in SV40-positive MM patients compared to that of SV40-negative MM patients showed that six cytokines, IL-15, EOTAXIN, MIP-1β, IL-18, IFN-α2, and HGF were up-regulated (p<0.01) whereas 2 cytokines, MCP-3 and MIF (p<0.01) were found at lower levels (Figure 4). In these patients the C-C chemokine RANTES although it did not show a significantly difference showed a high level of concentration in SV40 negative MM patients (34.000 pg/ml) compared with SV40 positive patients (18.000 pg/ml) reinforcing the association of this factor with asbestos. Conversely, the growth factor HGF, released in supernatant of transformed mesothelial cells was found, for the first time, significantly associated to SV40 infection in sera of patients with MM.

## Discussion

Malignant Mesothelioma is caused by a multi-step process arising from genetic alterations induced by asbestos fibers that drive the progressive transformation of normal mesothelial cells into MM [18], [32]. The hallmarks of asbestos fibres inhalation included early and sustained inflammation causally attributed to initial accumulation of alveolar macrophages promoting the subsequent generation of reactive oxygen species (ROS) that cause DNA damage and induce cells to proliferate in a chronic inflammatory milieu. A fair amount of evidence sustain the role of inflammation in inducing mesothelial cells to transcription and production of some cytokines, critical to the initiation of injury, fibrosis and tumor [26]. Thus, the enhanced ability of mesothelial cells to respond to asbestos fibers, oxidants and growth factors seem to be responsible for both dysregulation of mitogenic signaling and loss of tumor suppressor proteins that may govern MM pathogenesis.

In the present study soluble cytokines, basically linked to pulmonary inflammation, IL-1a, IL-3, IL-6, were found highly expressed in MM patients confirming recent experimental data reported by Hillegass and colleagues [28]. Conversely, in contrast to the study of Fox et al conducted in mesothelioma and mesothelial cell cultures derived from two different mouse strains, [29] sera from the Asb-workers contained significantly lower levels of GRO-α and no significant difference in serum concentration of GRO-α was found in MM with respect to the control group. This finding seems to indicate that GRO-alpha gene is regulated differently in humans compared to mouse strains probably due to the complexity and interspecies diversity of the chemokines. To note, in pleural fluid of MM patients we found GRO-α under detectable level, confirming our previously sera result (data not shown).

Notably, a new pattern of immune-modulator molecules, IL-12(p40), β–NGF, TNF-β, IFN-γ, CTACK and RANTES, were reported for the first time in serum of these patients, although IL-12(p40) and β–NGF have been already detected in the microenvironment of tumoral mesothelial cells [33], [34].

The uniqueness of the work herein is the demonstration that the C-C chemokine RANTES, is strongly and specifically associated to the asbestos exposure, as documented in our series of Asb workers free from lung or pleural alterations. A significant increased gradient of the soluble concentration of RANTES, was demonstrated through the analysis of the healthy controls, the Asb workers and the asbestos-induced MM patients, which value was detected at high level in all subjects found exposed to asbestos.

RANTES is a known chemotactic cytokine produced by many cell types of the immune system and by tumor cells which is involved in immune regulatory and inflammatory processes The role of RANTES as tumor growth factors in the recruitment of tumor associated macrophages, and in metastatic spreading or angiogenesis is recently reported for humans tumors [35]–[37]. A possible mechanism of action for this molecule may be inferred from the findings of a recent study reporting that the expression of RANTES by breast tumor cells results not only in monocyte migration to the tumor site but also in pro-tumorigenic activities of RANTES, that may contribute to disease progression. Thus, RANTES has been used as a prognostic indicator in both breast and cervical cancers showing that high levels of RANTES in these malignancies correlate with a poor clinical outcome [36], [38].

In this study, in order to prove the independent role of RANTES as a possible prognostic biomarker for asbestos associated diseases, the influence on cytokines network exerted by the cofactor SV40 was additionally evaluated in infected patients. A synergistic action between SV40 and asbestos fibers has been recently suggested [12], [39]–[41] showing human mesothelioma cells to be highly sensitive to SV40-mediated transformation, acting on the expression of cellular growth factors. It is possible that SV40 may induce a growth advantage of MM cells, inactivating both cell cycle regulatory proteins and inducing increased or inhibition expression of specific growth factors and cytokines [39], [42]. At the light of these findings, a significative panel of 8 inflammatory molecules not including RANTES, was found associated to SV40 infection, reinforcing data of the specific association of this chemokine with asbestos.

Of note, among these cytokines, a high level of the hepatocyte growth factor (HGF), released during tumor transformation of the mesothelial cells, was detected for the first time in sera of MM patients SV40 infected. This data seem supported recent in vitro study where, in SV40-positive malignant mesothelioma cells, the HGF receptor, the Met oncogene product, was activated promoting the cell-cycle progression into S phase, toward malignant transformation. This finding suggested that a limited number of SV40-positive cells may be sufficient to direct non-infected cells to malignant transformation by paracrine loop, thus enhancing the asbestos activity [43], [44], [45].

Despite the limitations due to the small sample size, this pilot study demonstrated a significant increased level of the chemokine RANTES both in a selected series of workers chronically exposed to asbestos and in patients with asbestos-induced MM suggesting an association of this C-C chemokine with asbestos exposure.

## Conclusion

A strong link has been established between exposure to asbestos, inflammation and increased risk for malignant mesothelioma. This study shows, for the first time, that serum level of CC-Chemokine RANTES is significantly associated to asbestos exposure. However, further studies are envisaged on larger groups of asbestos-exposed individuals to confirm the prognostic usefulness of this biomarker in predicting disease progression, as recently reported for the breast carcinoma.
